# Endocrine Disruptor Compounds—A Cause of Impaired Immune Tolerance Driving Inflammatory Disorders of Pregnancy?

**DOI:** 10.3389/fendo.2021.607539

**Published:** 2021-04-12

**Authors:** John E. Schjenken, Ella S. Green, Tenuis S. Overduin, Chui Yan Mah, Darryl L. Russell, Sarah A. Robertson

**Affiliations:** ^1^ Adelaide Medical School and The Robinson Research Institute, University of Adelaide, Adelaide, SA, Australia; ^2^ Priority Research Centre for Reproductive Science, Discipline of Biological Sciences, The Hunter Medical Research Institute, New Lambton Heights and the University of Newcastle, Newcastle, NSW, Australia

**Keywords:** endocrine disrupting compounds, reproduction, reproductive immunology, pregnancy, fetal tolerance, developmental origins of health and disease

## Abstract

Endocrine disrupting compounds (EDCs) are prevalent and ubiquitous in our environment and have substantial potential to compromise human and animal health. Amongst the chronic health conditions associated with EDC exposure, dysregulation of reproductive function in both females and males is prominent. Human epidemiological studies demonstrate links between EDC exposure and infertility, as well as gestational disorders including miscarriage, fetal growth restriction, preeclampsia, and preterm birth. Animal experiments show EDCs administered during gestation, or to either parent prior to conception, can interfere with gamete quality, embryo implantation, and placental and fetal development, with consequences for offspring viability and health. It has been presumed that EDCs operate principally through disrupting hormone-regulated events in reproduction and fetal development, but EDC effects on maternal immune receptivity to pregnancy are also implicated. EDCs can modulate both the innate and adaptive arms of the immune system, to alter inflammatory responses, and interfere with generation of regulatory T (Treg) cells that are critical for pregnancy tolerance. Effects of EDCs on immune cells are complex and likely exerted by both steroid hormone-dependent and hormone-independent pathways. Thus, to better understand how EDCs impact reproduction and pregnancy, it is imperative to consider how immune-mediated mechanisms are affected by EDCs. This review will describe evidence that several EDCs modify elements of the immune response relevant to pregnancy, and will discuss the potential for EDCs to disrupt immune tolerance required for robust placentation and optimal fetal development.

## Introduction

Endocrine disrupting compounds (EDCs) are defined by their potential to alter endocrine function through mimicking or blocking the actions of endogenous hormones (1, 2). Exposure to EDCs is considered a contributing factor in the increasing prevalence of common metabolic, neurological and inflammatory diseases. Male and female reproductive disorders, and a myriad of conditions including obesity, diabetes, non-alcoholic fatty liver disease, neurodevelopmental disorders, allergy, asthma, autoimmunity, and cancer, are all associated with EDC exposure (1). Alarmingly, the estimated human disease cost of EDCs in 2016 was 2.33% of GDP ($340 billion USD) in the USA and 1% of GDP ($217 billion USD) in Europe (3). Recent reports commissioned by the World Health Organization recommend greater investment in research to better understand the health impact of EDCs. Identified research goals include the development of comprehensive testing methods to detect EDCs, improved reporting mechanisms for chemical composition of products, and the need for more cross-disciplinary research to fully understand the impact on public and global health of EDCs contacted in everyday life (1).

EDCs are structurally and functionally diverse chemicals that can be natural or synthetic in origin (1). Natural forms include phytoestrogens found in widely-consumed food and animal products. These are likely less harmful than synthetic EDCs since they have generally low affinity for estrogen receptors (ER) (4), and exhibit low stability compared to many synthetic compounds that are engineered to be stable. However, given the high levels present in some foods, including infant formula, and the fact that the abundant phytoestrogen genistein binds ERβ with relatively high affinity, the potential health impacts of phytoestrogens need to be considered (4, 5).

Synthetic EDCs are far more diverse with several hundred identified and classified as persistent (exhibiting bioaccumulation) or non-persistent in the environment (2, 5). These compounds are present in many commonly used household and industrial products. They include chemicals used as solvents or lubricants, plasticizers, pesticides, fungicides, and pharmaceutical agents, that are present in plastics, detergents, household chemicals and building products, fire retardants, food, medicines, personal care products, perfume, and cosmetics (5).

EDCs interfere with the synthesis, biological actions, and metabolism of endocrine hormones, and disrupt hormone-regulated homeostatic processes in many tissues and physiological systems (2, 5). Through competitive interactions with hormone receptors, EDCs can act as agonists or antagonists and have a multitude of effects that range from enhancement, dampening, or blocking the action of endogenous hormones (2). Depending on the nature of the interaction, EDCs often exert non-monotonic dose responses characterized by low-dose effects, rather than linear dose responses like most other bioactive agents (6, 7). EDCs can also modulate synthesis of hormones and their respective receptors (2). Through these actions, they can interfere with physiological events and tissue homeostasis over the entire life cycle (2, 8–10). Depending on variables such as the duration, type, and dose of exposure, EDCs can exert transient or permanent impacts, to elevate long-term risk of chronic metabolic, neurological and immune diseases that may only become evident in later life (2, 9).

The events of reproduction, pregnancy, and fetal development are highly sensitive to EDCs because they involve a greater degree of tissue remodeling and hormone-dependence than other physiological processes. EDCs exert negative impacts on fertility and reproductive outcome, affecting gamete, embryo, and fetal development (2, 8, 11–13), with consequences that can cause fetal loss or attenuate offspring phenotype to impact lifetime health (2, 9, 14–19). The specific mechanisms by which different EDCs exert adverse developmental effects are not yet clear, and are likely to be complex and diverse. A large body of research has been generated in recent years to describe actions in different male and female reproductive tissue compartments. These actions are largely attributed to disruption of the hormone signaling that regulates most aspects of male and female reproductive physiology (2, 5).

In addition, EDCs are now understood to affect immune system development and function (20, 21), modulating many aspects of inflammatory and immune responses involving both the innate and adaptive immune compartments (22–29) (**Table 1**). Most reproductive processes are intimately dependent on a functional and appropriately balanced immune response (32, 33). Placental development and fetal growth are particularly dependent on adequate support from the maternal immune system (34), and a deficit in maternal immune cells and mediators that confer fetal tolerance is a central cause of poor gestational outcomes and impaired fetal development. An aberrant maternal immune response, that is insufficient in strength or skewed towards inflammation, can manifest as infertility, pregnancy loss, or a poor gestational outcome (32, 33, 35). Most often, these outcomes stem from failure of the maternal immune response to support embryo implantation and allow robust placental development (35–37).

**Table 1:** Common endocrine disrupting chemicals shown to impact the immune response.

Common Endocrine Disrupting Chemicals	Description/Sources
**Bisphenol-A (BPA)**	* most pervasive EDC
	* estrogen mimic
	* found in canned food, dental sealants and composites, and widely used in manufacture of epoxy, polycarbonate plastics and unsaturated polyester resins (30)
**Phthalates**	* widely used as plasticizers in polyvinyl chloride (PVC) products to impart flexibility and durability, including building materials, toys, personal care products and medical devices (31)
	* gained considerable attention due to specific concerns about pediatric exposure (31)
**Alkylphenols** * Nonylphenol (NP) * Octylphenol (OP)	* widely used as non-ionic surfactants in household applications, industrial and cosmetic products
	* undergo significant bioaccumulation due to their lipophilic properties and have weak estrogenic activity
**Butyltins** * Tributyltin (TBT) * Dibutyltin (DBT)	* found in plastic food containers, plastic water bottles, PVC pipes
**Insecticides** * Dichlorodiphenyltrichloroethane (DDT)	* agricultural and household use
	* persists in environment
	* estrogen mimic
**Fungicides** * Vinclozolin	* agricultural and household use
**Herbicides** * Atrazine	* agricultural use on crops
	* used on artificial turf
**Parabens** * Methylparaben	* common preservatives
	* used in food, cosmetic and pharmaceutical products
	* estrogenic effects
**Brominated flame retardants** * Polybrominated diphenyl ethers (PBDE)	* flame retardant used in household and industrial products
	* endocrine disrupter with carcinogenic properties
**Synthetic hormones** * 17α-ethinylestradiol	* used in oral contraceptive pills and found as a contaminant in wastewater
	* strong estrogenic properties

These considerations raise the question of whether the adverse effects of EDCs on reproduction and pregnancy are at least partly due to mechanisms mediated by immune cells. Given the central role of the immune response in pregnancy, and the ubiquitous exposure of humans to environmental EDCs, it seems likely that EDC-induced immune disorders are a factor in the increasing incidence of fertility and gestational disorders.

This review will summarise evidence that common EDCs have capacity to interfere with pregnancy and fetal development through modifying maternal immune cells and mediators. We make the case that, given its critical importance to pregnancy outcome, and its sensitivity to perturbation by EDCs in other settings, the immune response warrants investigation as a mechanism by which EDCs affect reproductive success. Ultimately, devising strategies to protect humans and animals from the adverse reproductive effects of EDCs will require greater understanding of the how the immune system–EDC interaction contributes.

## Endocrine Disrupting Compounds and Reproduction

EDCs are well-documented to interfere with male and female reproductive hormone function, through genomic and non-genomic mechanisms that exert a wide range of endocrine disturbances (5). In particular, these chemicals interfere with binding of hormones to their corresponding receptors, notably including estrogen receptor and androgen receptor to cause either agonistic or antagonistic effects. The net consequence is interference with physiologically normal signal transduction pathways, eliciting downstream changes to target gene expression and cellular function (38). Different EDCs exert a variety of effects on hormone signaling depending on the timing of exposure and the amount of EDC administered (2, 5).

Dysregulated hormone synthesis and signaling in reproductive tissues, and systemically in the hypothalamic-pituitary axis, thyroid, and other tissues influencing reproductive function, converge to have substantial consequences for sexual maturation and fertility (39–41). Emerging evidence indicates that sensitivity to EDCs is modulated by age and a range of environmental, lifestyle, and genetic factors that can exacerbate the impact of EDCs on reproductive health (3, 42, 43). These factors contribute to the difficulty in comparing studies and considerable discrepancies between study outcomes (3, 44).

Large clinical studies show correlations between EDC exposure and fertility disorders in women. Most notably, occupational exposure to EDCs, or consumption of EDC-laden foods, are associated with increased risk of infertility, time-to-pregnancy, and early pregnancy loss (14, 45). These effects may reflect early life and life course accumulated exposures. In particular, prenatal effects of EDCs are linked with later life incidence of reproductive conditions including polycystic ovarian syndrome, endometriosis, uterine fibroids, and reproductive cancers (9). In an IVF setting, women exposed to certain pesticides appear more likely to exhibit defects in oocyte maturation and developmental competence, leading to impaired fertility, embryonic defects, and poor IVF outcomes (41, 45).

Research in rodent models provides insight on how EDCs impact reproductive endocrinology (2, 40, 46). These manifest most obviously as altered timing of sexual maturation, impaired gamete development, and reduced fecundity (39, 46). For example administration to rodents or large animals of plasticizers such as phthalates and bisphenol A, (BPA), or pesticides including vinclozolin and glyphosate, all cause reduced ovarian weight, impaired follicle growth and oocyte viability, and reduced synthesis of ovarian sex steroid hormones (46–48). For detailed information on the specific impacts of EDCs on female reproductive physiology, the reader is directed to the following reviews (2, 5, 9, 40, 46, 49).

EDCs also exert considerable effects on male reproduction and gamete developmental competence. Direct or gestational exposure of male rats and mice to any of several EDCs leads to reduced reproductive capacity, characterized by decreased gonad weight, testosterone levels, and gamete quality, as well as increased likelihood of reproductive conditions including testicular cancer, cryptorchidism, and hypospadias (39, 40, 50). *In vitro* studies show in cattle that exposure to low doses of the herbicide atrazine reduces sperm viability and impairs capacity to undergo acrosome reaction in response to calcium signals (51). In men, epidemiological evidence shows a clear negative association between EDCs and male reproductive parameters, in association with reduced sperm concentration, motility, viability, DNA integrity, and altered sperm methylation patterns (40, 50, 52, 53). Various EDCs are also readily detectable in seminal plasma (54), and the seminal vesicles, which are the major source of seminal plasma, are an important target of EDCs including diethylstilbestrol that targets estrogen receptor-α (55). These changes are likely to compromise fertility, and alter reproductive outcomes beyond the fertilising capacity of sperm. In men utilising IVF clinics, exposure to phthalates was associated with differential methylation of specific DNA sequences in sperm, and was inversely associated with blastocyst quality (53). A wide range of specific effects of EDCs on male reproduction are reported, and these are reviewed in detail elsewhere (2, 5, 9, 39, 40).

## Endocrine Disrupting Compounds and Pregnancy

Fetal and placental development are highly hormone-dependent processes and are therefore particularly susceptible to endocrine signaling disturbances (56–58). Reproductive-aged women are at high risk of EDC exposure, especially through everyday exposure to personal care products and household chemicals, and the events of pregnancy would reasonably heighten the health risks of EDC exposure in women (56). There is compelling evidence implicating EDC exposures as a risk factor in a range of pregnancy disorders (59–63). Several clinical and epidemiological studies link EDCs, notably pesticides and plasticizers, in common pregnancy complications that together affect around 20% of women, including recurrent miscarriage, fetal growth restriction, preeclampsia and related hypertensive disorders, and preterm birth (13, 56, 61, 64, 65). Many studies consistently show a wide array of EDCs are detectable in the urine, cord blood, plasma, amniotic fluid and breast milk of the vast majority of pregnant women (66–68). Patterns of exposure depend on geographic, socioeconomic, occupational and lifestyle factors, and fluctuate over the course of pregnancy, to occur in infinitely variable combinations (known as the ‘exposome’) that might have stronger relationships to adverse outcomes than any individual chemical exposure (69, 70). Nevertheless, while causal relationships are difficult to prove in humans, extensive studies show strong evidence of correlations between adverse clinical outcomes and serum or urinary levels of bisphenol A (BPA), phthalate metabolites, organophosphate pesticides, and other EDCs (61, 71–73).

EDCs may operate through pre-pregnancy exposures that affect organs systems critical for pregnancy health, through gestational exposures that interfere with hormone control of fetal and placental development and function, or other *via* systemic adaptations required to sustain pregnancy (63). There is clear evidence that pregnant women with existing health disparities, associated with low socioeconomic status, or certain racial groups such as non-white women in the US where levels of chemical toxicants are often higher, exhibit a disproportionate health burden associate with EDC exposures (3, 44).

The placenta is implicated as an important target for EDC actions. As a rapidly developing, dynamic organ the placenta is highly responsive to hormone regulation during its morphogenesis, and expresses a wide array of hormone receptors that control placental supply of nutrients to the growing fetus (57, 58). The placenta adapts to fetal and environmental cues to reconcile fetal demand for growth with nutrient availability, and disruption of hormone signaling interferes with this adaptive capability to disturb fetal growth and developmental programming (57).

Animal models document a range of potential mechanisms by which EDCs disrupt placental and fetal development. Some EDCs, notably including BPA and triclosan, accumulate directly in placental tissues, where they modulate placental hormone synthesis and metabolism (74, 75). *In vitro* experiments show that a range of EDCs can exert direct effect in trophoblasts including regulation of signaling pathways to cause genetic and epigenetic changes that impact cell survival and invasive capability (75). It seems likely that effects of EDCs are prominent in early pregnancy during placental morphogenesis, when the extent of invasion into maternal tissues, and interaction with the maternal vasculature, is rate-limiting for later gestation placental transport function (58). However because EDC effects in placental cells have not been well investigated to date, it is not yet possible to discern the contribution of direct effects in trophoblasts, versus mechanisms that involve the maternal compartment (58).

## Endocrine Disrupting Compounds and Offspring Health

The effects of EDCs on the developing fetus have a lasting impact on offspring phenotype and susceptibility to later life health and disease (9, 76). The developmental defects caused by maternal EDC administration in pregnancy can have life-long and even transgenerational consequences (77). Maternal EDC exposures likely impart changes to offspring health and behaviour through direct effects in the placenta and fetus, as well as indirectly through maternal physiological adaptations required to support pregnancy.

Animal studies show EDCs including pesticides, phthalates and BPA act to decrease fertility, alter anogenital distance, cause early puberty, and disrupt testis/ovarian function in both male and female offspring (40). These exposures not only disrupt offspring reproductive capacity, but also alter aspects of development affecting brain and endocrine function (15, 16, 78). In humans there is compelling evidence that gestational exposure to a variety of EDCs during fetal life leads to decreased infant birth weight, reduced anogenital distance in male neonates, increased incidence of childhood obesity, and alterations to neurodevelopment and cognitive function, leading to reduced IQ and behavioral problems (9).

Concerningly, there is emerging evidence that EDCs can exert transgenerational effects, such that not only the immediate offspring, but also future generations may be impacted after maternal contact in pregnancy (9, 40). This may be mediated through epigenetic modifications to DNA methylation profiles in fetal gametes, caused by inappropriate timing or inhibition of activation signals during gamete development, or through DNA adduction induced by EDCs or their metabolites (40, 79).

The impact of paternal exposures and their mechanisms of action are less well defined, but emerging evidence points to effects on offspring phenotype mediated by altered epigenetic properties of sperm (80). Furthermore, EDCs present in seminal plasma (54), or altered seminal plasma composition resulting from EDC-attenuated ERα signaling (55), have potential to transmit effects of paternal exposures to offspring. This could occur by impaired capacity of seminal plasma to support sperm integrity, or by attenuating seminal plasma signals that modulate female reproductive tract gene expression and receptivity for pregnancy (81, 82). In mice, it has been reported that paternal contact with BPA prior to conception impairs offspring spatial memory (19), and alters social behaviour with increased anxiety in male offspring (83). These can begin very early in the life course – male fetuses exposed *in utero* to the fungicide vinclozolin or pesticide dichlorodiphenyltrichloroethane (DDT) exhibit later epigenetic changes in sperm that can be transmitted to male offspring (84). In zebrafish, exposure to synthetic estrogen 17α-ethinylestradiol leads to an altered sperm and testicular transcript content, causing lymphodema in offspring (85). In human, recent evidence from a large-scale epidemiological study demonstrates a link between birth defects and fathers’ occupational exposure to EDCs (17).

## Viviparous Reproduction and the Immune Response

The embryo and the gestational tissues formed after implantation express antigens foreign to the mother, including transplantation antigens encoded by major histocompatibility complex (MHC) genes (33, 86). Both the innate and adaptive compartments are involved in the maternal immune adaptions required to avert effector immune responses to conceptus antigens (86, 87). Contrary to common assumptions, pregnancy requires a state of adaptive immune tolerance that depends on maternal lymphocytes being actively primed to recognise conceptus antigens (35, 86). Priming of the adaptive immune compartment must commence prior to implantation in order to initiate the necessary events of implantation, placental development and fetal growth, and ultimately to orchestrate on-time parturition and birth (35, 86).

### Immune Mechanisms Essential for Implantation and Placental Development

Tightly controlled maternal immune regulation is important over the course of pregnancy, but the most critical period is the peri-conception phase spanning fertilization to embryo implantation (35). A series of dynamic changes in the uterine immune response determine whether or not embryo implantation can occur (88), and are instrumental in setting the trajectory of fetal development and shaping the offspring phenotype (35, 80, 89). Immune adaptation commences with sex hormone-induced changes in the ovulatory cycle followed by an inflammation-like response to seminal fluid components at coitus (90). Estrogen and seminal fluid together induce an influx of neutrophils, macrophages and dendritic cells (DCs), into the mucosal surface of the cervix and uterus (91–94). This is followed by transition to an anti-inflammatory and pro-tolerogenic immune environment in order to acquire embryo receptivity (34, 35, 86). Implantation only occurs if immune cells in the uterine endometrium exhibit a favourable, permissive response. In particular, expansion and recruitment of specialized immune cells known as regulatory T cells (Treg cells) must occur (95–98). Treg cells interact with dendritic cells and macrophages to promote decidualisation of uterine stromal cells, suppress inflammation, and inhibit effector immunity towards fetal antigens.

**Figure 1:**
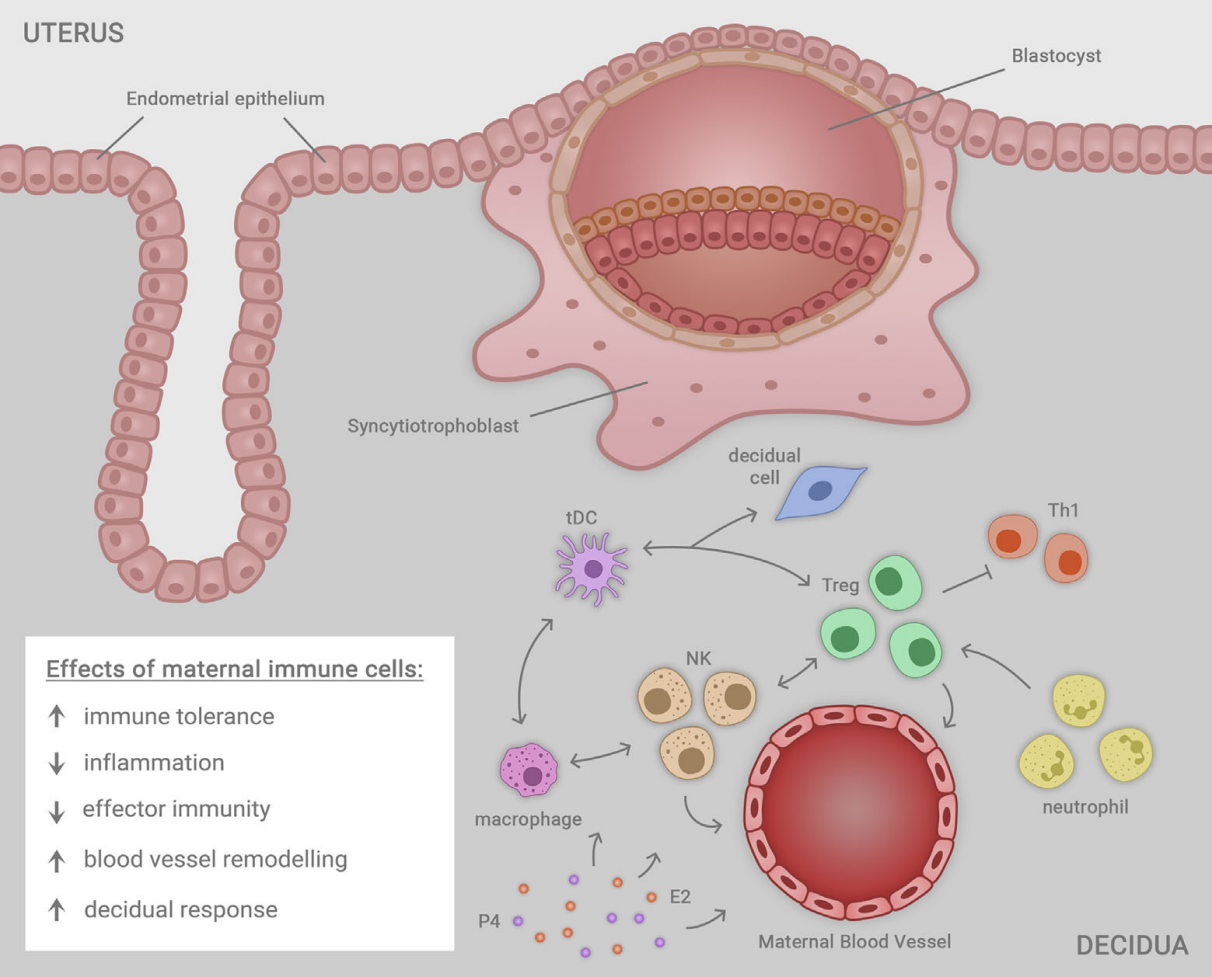
Immune cells including macrophages, natural killer (NK) cells, regulatory T cells (Treg cells), neutrophils and tolerogenic dendritic cells (tDC) residing in the uterine decidua each contribute in a network of cellular interactions to facilitate embryo (blastocyst) implantation and trophoblast outgrowth, required for progression to healthy pregnancy. The decidual immune cells exert a range of regulatory effects on the local microenvironment that each contribute to the success of implantation, ensuring robust placental development that in turn supports healthy fetal growth and development in later gestation. The immune cells together act to mediate immune tolerance, suppress inflammation, inhibit effector immunity mediated by T helper type 1 (Th1) cells, promote uterine blood vessel remodeling, and facilitate transformation of uterine stromal cells in the decidual response. Ovarian sex steroid hormones estrogen (E2) and progesterone (P4) act to regulate immune cell populations through direct effects in immune cells, and indirect effects mediated by non-immune cell synthesis of immune-regulatory factors.

After implantation, an array of soluble mediators including cytokines, chemokines, steroid hormones, and prostaglandins released from placental trophoblasts are important for sustaining the developing fetal-placental unit (32). As well as Treg cells, abundant populations of uterine natural killer (uNK) cells act to mediate structural changes in the decidual vasculature that support placental invasion and development (99–101). Macrophages, DCs, and Treg cells each interact with uNK cells to facilitate the uterine vascular changes, while continuing to suppress inflammation and prevent immune effector cell activation (35, 100, 101) (**Figure 1**).

## Endocrine Disrupting Compounds and the Immune Response to Pregnancy

Maternal EDC exposure is an identified risk factor in unexplained infertility and pregnancy complications, including preeclampsia, intra-uterine growth restriction, recurrent miscarriage, and spontaneous preterm birth (13, 61, 64, 65). Interference in hormone synthesis and signaling is implicated in the mechanisms by which EDCs contribute to pregnancy disorders (9), and there is a strong biological rationale to implicate inflammation, oxidative stress, and immune cells as local mediators of the pathophysiological changes induces by hormone dysregulation (59). That immune and inflammatory mechanisms are central to infertility and pregnancy disorders supports the prospect that EDCs act, at least in part, by driving an inappropriate maternal immune response (20, 21).

There is some evidence that EDCs are especially problematic in the peri-conception phase of pregnancy, when the maternal immune response is first established and the critical events of implantation and early placentation occur. Elevated phthalate metabolites in urine were shown to correlate with altered progression of embryo implantation, as indicated by a slower or faster rise in human chorionic gonadotrophin, with different metabolites appearing to be protective or adverse in their effects (102). Whether immune mechanisms are involved is not known, but seems biologically plausible. Others have shown that first trimester maternal peripheral blood cytokine levels correlate with the presence of several EDCs in urine, with a notable association between phthalates and pro-inflammatory interleukin (IL)-8 and interferon (IFN) (70). In another study, clear associations between polybrominated diphenyl ethers (PDBEs) and pro-inflammatory cytokines IL-6 and tumor necrosis factor (TNF), as well as between per- and poly-fluorochemicals (PFAS) and IL-6, were found in maternal peripheral blood in the second trimester (103). Similar associations between EDCs and pro-inflammatory cytokines were seen at term, in infant cord blood (70). These observations are consistent with EDCs acting to impair resolution of the inflammatory response in early pregnancy and compromise tolerance as pregnancy progresses, but additional studies would be required to prove this.

Only a small number of mechanistic studies have specifically explored the impact of EDCs on maternal or fetal immune parameters in pregnancy, but several point to a pro-inflammatory pathology that affects the vascular adaptations required for robust placental development. In mice, short-term oral BPA exposure in early pregnancy was shown to cause impaired spiral artery remodeling and intra-uterine growth restriction (11). Although the number of uNK and mast cells was not changed, this study did not assess phenotypes of these cells, or the potential influence of other immune cell populations. Another study reported reduced trophoblast invasion and impairment of uterine vascular remodeling after low dose BPA administration in mice, along with preeclampsia-like features of maternal hypertension and elevated angiogenesis biomarkers and glomerular atrophy (104). Also consistent with an inflammatory mechanism, administration of polychlorinated biphenol (PCB) to mink resulted in uterine vascular changes and placental lesions, with degeneration of endothelial and trophoblast cells, particularly in the placental labyrinth zone (105). Low dose 17α-ethinylestradiol, used in oral contraceptive pills and prevalent in water supplies, caused impaired spiral artery remodeling, altered placental development, and fetal growth restriction (106). In non-pregnant mice, uterine expression of heat shock proteins (HSPs) that play important roles in antigen presentation and DC function are elevated in response to low dose BPA (107), but the impact of elevated HSPs on pregnancy is not clear.

Studies in other reproductive tissues are consistent with possible pro-inflammatory and immune-mediated effects of EDCs (108). In the mammary gland, BPA exposure in utero causes long term changes in expression of both pro- and anti-inflammatory cytokines, and this is postulated to be a potential mechanism for programming breast cancer risk (109).

As well as influencing the maternal immune compartment, EDCs likely elicit direct effects on immune cells in the placenta and fetus. The presence of EDCs in amniotic fluid and cord blood shows that many chemicals cross the placenta to access fetal tissues (58, 75). A wide range of EDCs including pesticides, plasticizers, fire retardants, and components of personal care products can be detected in the placenta (75). Compelling evidence of EDC effects on the developing fetal immune response is emerging (110, 111). In particular, phthalates and phenols are implicated as a factor in fetal programming of asthma and allergic airways disease, while heavy metals and air-borne particulates also contribute (21, 112). A wide range of immunomodulatory effects of EDCs on human immune cell development are reported, through mechanisms operating at the cellular, molecular, and epigenetic levels to alter innate and adaptive immune function in offspring (110).

## EDCs and Hormone Control of Immune Cells

A clear mechanism for EDCs exerting significant influence on the maternal immune environment exists, as endocrine signaling in immune cells is an important aspect of normal immune regulation (113). Steroid hormones exert both direct and indirect influence on immune cells, the former through ligation of classical steroid hormone receptors for estrogen, androgens, and progesterone, to regulate a wide range of target genes. In addition, steroid hormones have rapid non-genomic effects in immune cells *via* binding to non-classical receptors on the cell membrane or in the cytoplasm (114). As well, steroid hormones control expression of a vast array of cytokines and chemokines in non-immune cell lineages in hormone-responsive reproductive tissues, to exert indirect effects on resident immune cell populations through this route (115).

It is well known that female sex steroid hormones exert potent regulatory effects on immune cells systemically and locally within the female reproductive tract over the course of the menstrual cycle and during pregnancy. In particular, estrogen and progesterone play important roles in the induction of maternal immune tolerance, both through direct signaling in immune cells and indirectly through actions on epithelial and stromal cells in the female reproductive tract (116, 117). Over the course of the estrous and menstrual cycle and after conception, estrogen and progesterone are key factors in driving expansion of Treg cells in readiness to accommodate embryo implantation (118–120).

**Figure 2:**
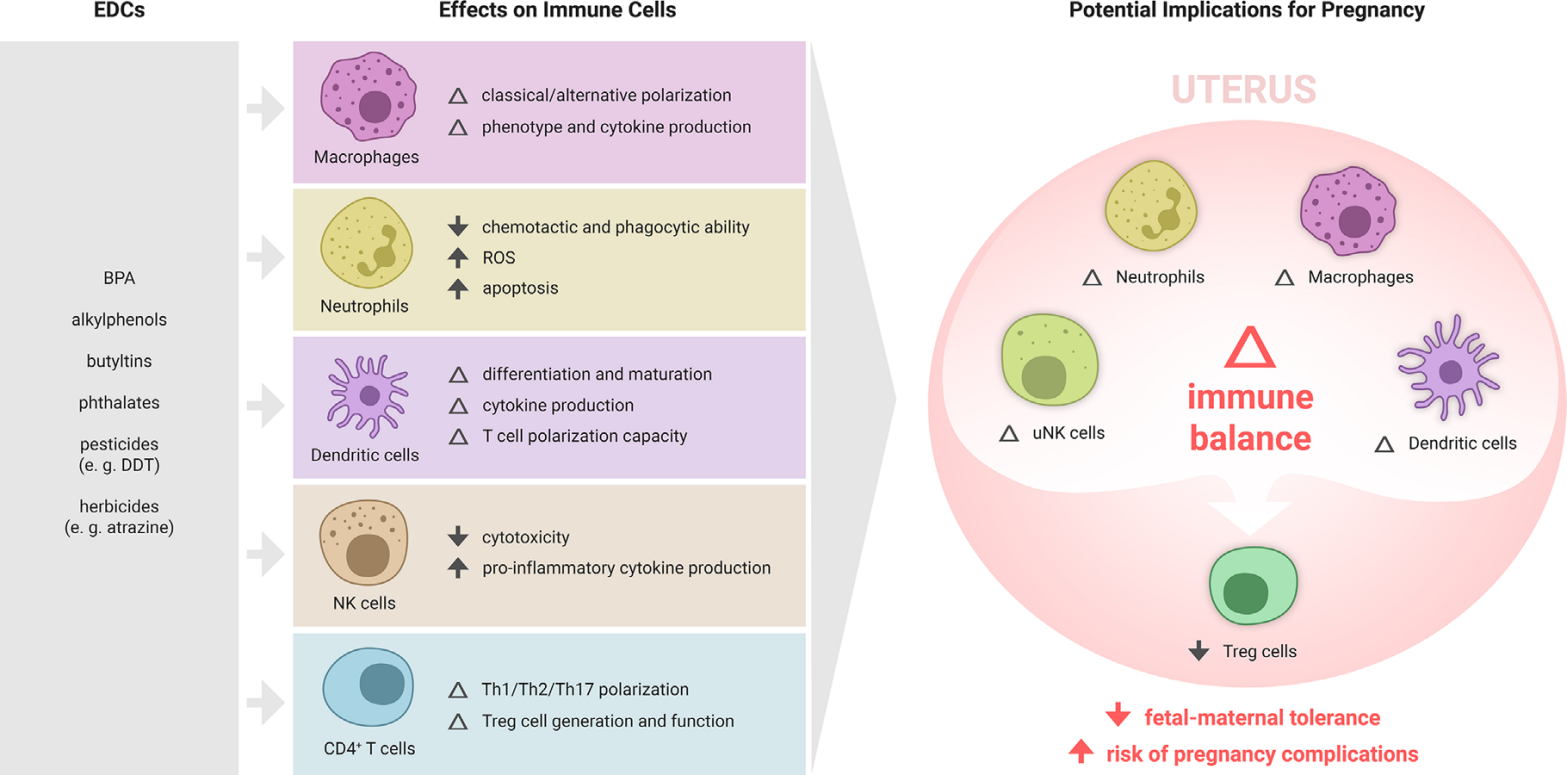
Summary of EDC effects on immune cell subsets and potential implications for maternal immune adaptation to pregnancy. Various EDCs affect the differentiation, phenotype and function of specific immune cell subsets, each of which play important roles in maternal immune adaptation to pregnancy. While the effects of EDCs on the immune response to pregnancy are yet to be formally examined, there is substantial evidence from other settings showing that various ECs can modulate macrophages, T cells, NK cells, and dendritic cells. In particular, EDCs that impair the generation of regulatory T cells (Treg cells), key mediators of fetal-maternal tolerance that are essential for embryo implantation and placental development, are likely to elevate susceptibility to pregnancy complications, and warrant investigation as contributing risk factors in recurrent miscarriage, preeclampsia, preterm birth and related gestational disorders.

Given this direct and indirect regulation by hormones, immune cells are highly susceptible to the effects of EDCs. EDCs broadly affect various immunological processes, including cellular and humoral responses, survival, differentiation and phenotypic maturation, as well as secretion of cytokines and other immune signaling mediators (22). Emerging evidence demonstrates substantial potential for EDCs to interfere with the endocrine signaling required for maternal immune adaptation to pregnancy (**Figure 2**). Below, we summarise the current evidence for EDC action on the innate and adaptive components of the immune response relevant to pregnancy, with a focus on immune cell types affected by EDCs and implicated in reproductive success. The argument that EDCs may act in pregnancy through influencing the maternal immune response is supported by studies of EDC effects on immune cells in other tissue settings and disease contexts.

### Macrophages

Macrophages contribute to embryo implantation, placental development, and timing of birth (35, 121, 122). In the maternal compartment, immune-regulatory macrophages constrain inflammation, influence the adaptive immune response, and modulate uterine vascular function (123). EDC disruption of their functional phenotypes is likely to adversely impact placental morphogenesis, pregnancy progression and fetal development. In the placenta, a large population of fetus-derived macrophages known as ‘Hofbauer cells’ exert direct effects on placental development and transport function. These cells can respond to proinflammatory stimuli and contribute to placental inflammation (124), so would also be susceptible to immune-modulatory effects of EDCs. Placental macrophages have been shown to upregulate production of prostaglandin E2 (PGE2) and cyclo-oxygenase-2 after exposure to mono-2-ethylhexyl phthalate (MEHP), the active metabolite of diethylhexyl phthalate (DEHP) (125).

Evidence from animal studies indicates that EDCs have capacity to alter macrophage phenotype and function, in a manner dependent on the polarization state of the macrophages at the time of exposure and the specific EDC (110). The most extensive evidence exists for effects of EDCs in M1-like classical macrophages. In murine macrophages, treatment with BPA, the alkyl phenols p-n-nonylphenol (NP) and p-n-octylphenol, or the chlorinated phenols 2,4-dicholophenol and pentachlorophenol, each lead to inactivation of nuclear factor kappa-light-chain-enhancer of activated B cells (NF-κB) signaling and suppression of TNF and nitric oxide (NO) following stimulation with lipopolysaccharide (LPS) (126–130). Interestingly, the capacity of BPA to suppress LPS-induced macrophage polarization is blocked by the ER antagonist ICI 182.780, suggesting BPA acts to regulate NF-κB signaling *via* ER (128, 129). Some of these effects occurred independently of classical ER signaling and were likely mediated by non-classical ER (129).

Other studies report that BPA and other EDCs have differing effects on macrophage production of pro-inflammatory cytokines and mediators, promoting a more activated, classical M1-like phenotype. For example in the mouse, benzo(a)pyrene (B(a)P) and hexachlorobenzene increase the production of NO in macrophage cell lines (127, 131). Similarly, human THP1 cell line-derived macrophages cultured with BPA exhibit increased pro-inflammatory TNF and IL-6 expression, dependent on classical ER signaling (132) Finally, treatment of mouse macrophages with EDCs including BPA, NP, dicyclohexyl phthalate and B(a)P causes cell death through apoptosis and necrosis pathways (127, 131).

Recent studies indicate impacts of various EDCs on M2-like alternative macrophages. In mice, exposure to polybrominated diphenyl ethers (PBDE) enhances estrogen mediated regrowth of mammary glands, in a manner potentially mediated by enhanced IL-10 expression and polarization of macrophages towards an M2-like state (133). Similarly, *in vivo* oral exposure of mice to BPA promotes the transition from ductal carcinoma *in situ* to invasive breast cancer through increases in pro-tumorigenic cluster of differentiation (CD)206^+^ M2-like alternatively activated macrophages (134). In contrast, other studies demonstrate that *in vitro* treatment of NP to mouse bone marrow-derived macrophages decreases their polarization by IL-4 toward an M2-like phenotype, associated with reduced survival in LPS-induced sepsis (135).

These studies indicate EDCs at physiological doses may promote or inhibit several aspects of both classical and alternate macrophage activation and effector function. The differential effects observed are likely due to differences in dose, context and type of EDC, and therefore, further work is required to develop greater understanding of the effect of EDCs on macrophages in various settings in mice and humans.

### Neutrophils

Neutrophils are important in preparing the female reproductive tract for embryo implantation, especially after coitus when they clear microorganisms, seminal fluid debris, and superfluous sperm, and help guard against sexually transmitted infection (90). Recent studies in mice and humans show that neutrophils are programmed by decidual signals to acquire an activated, pro-angiogenic phenotype (136, 137) akin to functions observed for tumor-associated neutrophils in cancer (138), suggesting a key role for neutrophils in establishing pregnancy. Given their importance in protecting from infection, and their emerging roles in regulating decidualization and placental development, studies to understand the impact of EDCs on uterine neutrophils may reveal novel pathways that exert long term influence on offspring health.

In both animal and human models, various EDCs impair neutrophil chemotactic and phagocytic ability and increase neutrophil apoptosis (139–142). In humans, chronic exposure to the pesticide DDT leads to a reduction in neutrophil chemotactic and phagocytic capacity that inversely correlates with incidence of infectious disease (143). BPA exposure is associated with increased reactive oxygen species (ROS) in human neutrophils *via* ER signaling, but does not cause changes in ROS-dependent formation of neutrophil extracellular traps (139).

### Dendritic Cells

Several effects of EDCs on DC differentiation and maturation are reported, where EDCs have been shown to shift the polarization and expression of maturation markers on DCs. In murine models, *in vitro* atrazine exposure leads to phenotypic changes, causing a dose-dependent loss of DC surface MHC class 1, as well as decreased CD86, CD11b, CD11c and CD14 expression (144). Other studies show EDCs alter DC cytokine production in mice, eliciting increased TNF and decreased IL-10 (145, 146). In other studies, BPA and NP induce the differentiation of murine bone marrow cells into DCs, with BPA having a more substantial effect than NP in altering differentiation capacity (147).

Mechanistic studies show that EDCs exert effects on DCs through both ER-dependent and –independent pathways. In a model of ovalbumin-induced allergic lung inflammation, NP-treated mice developed more severe inflammation compared to the control, however this effect was eliminated in mice carrying an aryl hydrocarbon receptor (AhR) mutation, suggesting NP may affect DCs *via* AhR-dependent (ER independent) pathways (146). In humans, exposure to the alkylphenols NP enhance TNF and suppress IL-10 and type 1 IFN production in peripheral blood mononuclear cell (PBMC)-derived plasmocytoid DCs. The ER antagonist ICI 182.780 could reverse NP-induced TNF and IFN-β expression but was unable to reverse the suppressive effect of NP on IL-10 or IFN-α expression in plasmocytoid DCs, suggesting both ER-dependent and -independent pathways of alkyphenol regulation of DCs occur (145).

DCs are critical regulators of the strength and quality of an adaptive immune response through signals delivered at antigen presentation, and the impact of EDCs on DC antigen presentation has been explored. EDCs such as BPA influence DC maturation and phenotype leading to an increased capacity to induce T-helper (Th)2 responses (148). Similarly, suppression of type 1 IFNs in human plasmocytoid DCs by phthalates programs a Th2 phenotype in T cells characterized by suppressed IFN-γ and enhanced IL-13 production (149). In contrast, BPA exposure increases CD1α expression in human PBMC-derived DCs, enabling them to drive polarization of naïve CD4^+^ T cells towards a Th1 phenotype (150). While it is evident that EDCs can modulate DC phenotype, further studies are required to understand the specific effects of these DC changes for T cell phenotype, function, and maturation state.

Effects of EDCs on the DC contribution to pregnancy tolerance are unclear, but reasonably it would be expected that altered DC function and interaction with T cells could disrupt normal immune balance during pregnancy, and potentially skew permissive Treg cells towards destructive Th1 cells (151). In particular, an increase in TNF and IL-6 secretion by DCs in the peri-implantation period may create excessive inflammation that negatively influences embryo development. Whether EDC-exposed DCs inhibit Treg cells is an important question with substantial implications for fetal-maternal tolerance (151).

### Natural Killer Cells

Natural killer (NK) cells are affected by a wide range of EDCs, all of which appear to decrease NK cell recognition of and cytotoxicity towards tumour cells, even after brief and low concentration EDC exposure (110). These functions are elicited through changes in NK cell surface markers and production of inflammatory cytokines, ultimately leading to changes in cellular function (110). For example, tributytlin (TBT) and DDT exposure significantly decrease the cytotoxic function of human NK cells *in vitro*, modulating their expression of cell surface proteins including CD16, CD18 and CD56, as well as cytolytic proteins such as perforin and granzyme B (152, 153). The loss of NK cell lytic function following exposure to these EDCs appears to result from activation of protein kinase C and the mitogen-activated protein kinase pathway (154–156). However, not all EDCs elicit the same functional effects in NK cells. *In vitro* atrazine exposure inhibits the ability of NK cells to lyse target cells through blocking lytic granule release, without impacting the release of perforin or granzyme proteins (157), demonstrating that EDCs have differing functional effects, presumably reflecting different mechanisms of action.

EDCs also have significant impact on the production of inflammatory cytokines by NK cells, with several studies clearly demonstrating NK cells exhibit non-monotonic dose responses (110). Inflammatory cytokines such as TNF, IL1-β, IL-6 and IFN-γ were increased in response to low-dose exposure to various EDCs including TBT and dibutylin (DBT) (158–160). In the case of TBT-induced pro-inflammatory cytokines this was mediated through the activation of extracellular-signal-regulated kinase 1/2 and p38 kinase pathways (158–160).

Overall, these studies demonstrate that EDCs have substantial capacity to modulate NK cells, in ways relevant to uNK cell function in pregnancy. uNK cells are highly regulated by ovarian E2 and P4, and contribute to cyclic remodeling of the uterus over the course of the menstrual cycle in preparation for embryo implantation (161). Indeed uNK cells are the most abundant immune cell population in the uterus, where they promote decidualization, facilitate spiral artery remodeling, and play critical roles in placental development (99–101). In particular, the effect of EDCs on NK cells is relevant to the common condition of endometriosis where exposure to phthalates and PCBs are implicated, and altered uNK cells are reported (162, 163). To date, there are no studies examining specific changes to the phenotype or function of the uNK cell subset following exposure to EDCs, although experiments investigating effects of BPA on the uterine vasculature point to a possible role for uNK cells and a target of BPA effects (11, 101). Further research is required to examine the effect of EDCs on uNK cells and their role in mediating EDC effects on fertility and fecundity.

### CD4^+^ T Cells

In addition to indirect effects on T cell differentiation through the impact of EDCs on antigen presenting cells, there is evidence that EDCs directly influence CD4^+^ T cell differentiation and function. In studies of allergic disease, multiple EDCs have been shown to augment immunoglobulin (Ig)E-related responses through a common mechanism of enhancing T cell production of the Th2 inducing cytokine IL-4, *via* stimulation of nuclear factor of activated T-cells binding activity (30, 31, 164). Similar responses are observed in studies comparing adult versus prenatal exposure to BPA in male mice. In these studies, BPA promotes the antigen-stimulated production of Th2 cytokines (IL-10, IL-13 and IL-4) in adult mice, and both IFN-γ and IL-4 in adult offspring exposed to BPA prenatally (165).

*In vitro* studies of isolated mouse T cells exposed to EDCs from the alkylphenol family show suppression of Th1 development and enhanced Th2 development, in a manner independent of retinoic acid receptors, progesterone receptors, glucocorticoid receptor, retinoid x receptor, or ER (166). While this Th2 inducing capacity of EDCs is recapitulated in other studies (167, 168), it is notable that up-regulation of Th1 responses following either adult or prenatal exposure is also reported. Addition of BPA directly to splenocytes *in vitro* favours differentiation of Th1 cells, characterized by decreased IL-4 and increased IFN-γ production (169). The reason for variable effects of different EDC interventions on CD4^+^ T cell polarization remains unclear, but likely reflects the effects of different chemical entities, dose, duration or *in vivo* timing of exposure. Further studies are therefore required to fully understand the range of impacts of EDCs on CD4^+^ T cell differentiation.

Taken together, the published findings provide clear evidence that common EDCs such as BPA and phthalates can modulate T cell differentiation and function. The disturbance to Th1/Th2 polarization induced by EDCs may predispose to a range of inflammatory diseases (i.e. allergy, autoimmunity, and asthma) (110), and is also relevant to generation of maternal immune tolerance in pregnancy, where a specific suppression of Th1 cells is critical (34, 35).

As well as affecting Th1/Th2 polarization, BPA exposure influences the differentiation and functional phenotype of Treg cells. The elevated antigen-dependent induction of Th2 cytokines after BPA exposure seen in adult mice occurs in conjunction with a shift away from Treg cell generation. A dose-dependent decrease in the CD4^+^CD25^+^ Treg cells among CD4^+^ T cells with increasing BPA concentrations is reported (165). Similarly, BPA exposure during gestation and prior to weaning leads to a perturbed induction of oral tolerance characterized by a diminished accumulation of Treg cells (170). NP exposure in mice elevates Th2 and suppresses Treg cell numbers, which counteract the effects of ER agonists in the treatment of allergic rhinitis (171). In contrast, *in vitro* exposure of murine T cells to atrazine inhibits CD4^+^ T cell proliferation and elicits increased Foxp3^+^ Treg cells (172), again highlighting the variable effects of different EDCs on lymphocyte biology.

A key mechanism implicated in EDC modulation of Treg cells involves the transcription factor AhR. In mouse T cells, AhR is a key regulator of T cell differentiation into Treg and Th17 cells (173), and AhR activation promotes differentiation of functional Treg cells (174). EDCs such as dioxins can bind with high affinity and activate AhR, leading to the induction of functional Treg cells that suppress experimental autoimmune encephalitis (173). Activation of AhR by naturally occurring activators of the AhR signaling pathway, such as 6-formylindolo[3,2-b]carbazole, elicits an opposite effect where Treg development is suppressed and Th17 differentiation boosted (173). Since other EDCs including phenols and phthalates also affect immune processes *via* AhR modulation, the AhR pathway may be a central determinant of the differential impacts of different EDCs on T cell differentiation (175).

Given these effects, it seems highly plausible that BPA exposure affects the expansion of Treg cells in early pregnancy and has potential to compromise fetal-maternal tolerance. Other EDCs demonstrated to interfere with Treg cell populations could reasonably also impair maternal immune adaptation to pregnancy. Given that Treg cell insufficiency is implicated in a wide range of gestational disorders (35), this warrants investigation as a convergent mechanism by which EDCs contribute to elevated susceptibility and the rising incidence of these conditions.

## Conclusions

There is mounting evidence pointing to a contribution of EDCs in adverse pregnancy outcomes, as well as infertility and subfertility. While many studies have assessed mechanisms involving endocrine impacts of EDCs on reproductive processes, there has been limited exploration of mechanisms involving immune cells. Given the critical significance of the maternal immune response in pregnancy and the now substantial literature demonstrating that common EDCs interfere with key elements of the immune response relevant to pregnancy (**Figure 2**), it is important to consider immune dysregulation amongst the effects that EDCs may exert. In particular, EDC exposures in women prior to or around the time of conception have potential to disturb generation of maternal immune tolerance required at embryo implantation (34, 35), with ongoing consequences for placental morphogenesis, and susceptibility to gestational conditions that arise from compromised placentation. Since seminal fluid factors contribute to priming immune tolerance towards paternal antigens in women, is possible that male EDC exposures can also interfere with maternal immune tolerance in the female partner.

Research to uncover the significance of immune effects of EDCs in reproduction and pregnancy is aligned with the World Health Organization’s recommendation to improve knowledge on EDCs and human health (1). This research should span a range of approaches. Laboratory animal studies will be critical for demonstrating causal effects, elucidating mechanisms, and defining effects of frequency and strength of different EDC exposures. Future studies must be designed with a view to their translational impact for health and clinical relevance. For example, several studies to date have utilized supraphysiological doses of EDCs in order to demonstrate an impact, and these now need to be replicated using environmentally relevant doses (2). Nevertheless, there is compelling evidence from both reproduction (2) and immune (22, 110) studies that EDCs can exert substantial effects at low doses relevant to those in human environments. Building the evidence for environmentally relevant exposures is a priority, as is unravelling the complex biology of the U-shaped dose response curve typical of many EDCs (2).

Large scale human cohort studies will be important for investigating how EDCs interact with other environmental and lifestyle factors that attenuate their biological effects, and quantifying the relative risk attributable to EDC exposures. As noted in the US Endocrine Society’s Second Scientific Statement (2), studies must be carefully designed to take into account variables that likely attenuate EDC risk, including genetic diversity, socioeconomic status, geographic variables, age at exposure, and occupation (3, 42, 43). Importantly, pregnant women must be included in population studies and future research must focus on pregnancy as a critical period for investigation (56). In turn this work will inform public policy and justify government regulations on environmental exposures that impact reproductive and pregnancy health. The benefits will extend to rare and endangered species and economically important livestock animals, where EDCs will otherwise exert accumulating harm.

## Author Contributions

JS, EG, and SR assembled information and wrote drafts of the manuscript. TO, CM, and DR assembled information, reviewed drafts, and provided specialist insight. All authors contributed to the article and approved the submitted version.

## Funding

The authors acknowledge the funding support of the National Health and Medical Research Council (APP1099461, to SR) and Channel 7 Children’s Research Foundation (to SR and JS).

## Conflict of Interest

The authors declare that the research was conducted in the absence of any commercial or financial relationships that could be construed as a potential conflict of interest.
